# Dysregulation of heat shock protein 27 expression in oral tongue squamous cell carcinoma

**DOI:** 10.1186/1471-2407-9-167

**Published:** 2009-06-04

**Authors:** Anxun Wang, Xiqiang Liu, Shihu Sheng, Hui Ye, Tingsheng Peng, Fei Shi, David L Crowe, Xiaofeng Zhou

**Affiliations:** 1 Department of Oral and Maxillofacial Surgery, the First Affiliated Hospital, Sun Yat-Sen University, Guangzhou, PR China; 2 Center for Molecular Biology of Oral Diseases, College of Dentistry, University of Illinois at Chicago, Chicago, USA; 3 Research Institute & the Affiliated Hospital of Stomatology, Sun Yat-Sen University, Guangzhou, PR China; 4 Shanghai Children's Medical Center, Shanghai Jiao-Tong University, Shanghai, PR China; 5 Department of Pathology, the First Affiliated Hospital, Sun Yat-Sen University, Guangzhou, PR China; 6 Division of Epidemiology and Biostatistics, School of Public Health, University of Illinois at Chicago, Chicago, USA; 7 Graduate College, UIC Cancer Center, University of Illinois at Chicago, Chicago, USA

## Abstract

**Background:**

Recent proteomic studies identified Hsp27 as a highly over-expressed protein in oral squamous cell carcinoma (OSCC). Clinical studies that attempted to evaluate the prognostic values of Hsp27 yielded inconsistent results, which may be due to inclusion of OSCC cases from multiple anatomic sites. In this study, to determine the utility of Hsp27 for prognosis, we focused on oral tongue SCC (OTSCC), one of the most aggressive forms of OSCC.

**Methods:**

Archival clinical samples of 15 normal oral tongue mucosa, 31 dysplastic lesions, 80 primary OTSCC, and 32 lymph node metastases were examined for Hsp27 expression by immunohistochemistry (IHC). Statistical analyses were carried out to assess the prognostic value of Hsp27 expression for patients with this disease.

**Results:**

Dysregulation of Hsp27 expression was observed in dysplastic lesions, primary OTSCC, and lymph node metastases, and appears to be associated with disease progression. Statistical analysis revealed that the reduced Hsp27 expression in primary tumor tissue was associated with poor differentiation. Furthermore, the higher expression of Hsp27 was correlated with better overall survival.

**Conclusion:**

Our study confirmed that the dysregulation of Hsp27 expression is a frequent event during the progression of OTSCC. The expression of Hsp27 appears to be an independent prognostic marker for patients with this disease.

## Background

Oral squamous cell carcinoma (OSCC) is a complex disease arising in various sites, such as the oral cavity and oropharynx including the oral tongue and the base of the tongue. Tumors from these different sites have distinct clinical presentations and outcomes, and are associated with different risk factors [1] and genetic characteristics [2]. In this study, we focused on oral tongue (also known as mobile tongue), one of the most common sites for OSCCs. The oral tongue SCC (OTSCC) is more aggressive than other forms of OSCCs, with a propensity for rapid local invasion and spread [3]. The incidence of OTSCC is actually increasing in young and middle age groups [4-6].

Mammalian Hsp27 belongs to the family of proteins that display an enhanced expression in response to various stresses (e.g., heat shock). Several members of the Hsps family (e.g., Hsp90, Hsp70, Hsp27) have been implicated in tumorigenesis, which may be related to their ability to protect cells against spontaneous or stimulated programmed cell death [7]. The cytoprotective properties of the Hsps are closely linked to their primary functions as molecular chaperones. A critical function of Hsp27 is the ability to increase the resistance of cells to oxidative injuries. Hsp27 expression correlates with decreased levels of reactive oxygen species (ROS) and nitric oxide (NO^.^) [8-11].

The enhanced expression of Hsp27 has been associated with poor prognosis in several tumor types (e.g., gastric [12,13], liver [14] and prostate carcinoma [15]), but good prognosis in others (endometrial adenocarcinomas [16,17] and oesophageal cancer [18,19]). Several proteomic studies identified Hsp27 as a highly over-expressed protein in OSCC [20,21]. Our recent proteomic study also demonstrated that higher Hsp27 expression is associated with tongue SCC invasion and metastasis [22]. Several clinical studies have attempted to evaluate the prognostic value of Hsp27 for OSCC, yet yielded inconsistent results [23-28]. This apparent contradiction may partly be due to the inclusion of OSCC cases from multiple anatomic sites. In this study, we evaluate the prognostic significance of Hsp27 focusing on OTSCC, one of the most aggressive forms of OSCC.

## Methods

### Patients and tissues

The archived tissue samples from 80 cases of OTSCC, 31 cases of dysplastic lesions of the oral tongue and 15 normal tongue biopsies were utilized in this study. Clinical characterization of the OTSSC patients is summarized in Table 1. All OTSCC patients had received curative surgery (resection of the primary tumor and radical neck dissection). None of the patients had received any form of adjuvant therapy prior to their surgery. The tumor extent was classified according to the TMN system by UICC, and the tumor grade was classified according to the WHO classification of histological differentiation. Survival was calculated based on the date of surgery and the date of latest follow-up (or death). This study was approved by the ethical committee of The First Affiliated Hospital, Sun Yat-Sen University and the Institutional Review Boards (IRB) at University of Illinois at Chicago, IL.

**Table 1:** Clinical Characterization of the oral tongue squamous cell carcinoma (OTSCC) Patients*

		All OTSCCs (n = 80)	OTSCCs with follow-up data (n = 42)	Dysplastic lesions (n = 31)	Normal (n = 15)
**Age**	Median (Range)	48 (21–77)	46.5 (21–73)	51 (30–82)	47 (30–72)

**Gender **	Male: n (%)	43 (53.75)	20 (47.62)	17 (54.84)	6 (40.00)
	Female: n (%)	37 (46.25)	22 (52.38)	14 (45.16)	9 (60.00)

**Anatomic Site**	Tongue: n (%)	80 (100)	42 (100)	31 (100)	15 (100)

**Pathological T Stage**	Stage 4: n (%)	5 (6.25)	4 (9.52)		
	Stage 3: n (%)	14 (17.50)	8 (19.05)		
	Stage 2: n (%)	38 (47.50)	21 (50.00)		
	Stage 1: n (%)	23 (28.75)	9 (21.43)		

**Pathological N Stage**	Stage 2: n (%)	13 (16.25)	8 (19.05)		
	Stage 1: n (%)	19 (23.75)	12 (28.57)		
	Stage 0: n (%)	48 (60.00)	22 (52.38)		

**Clinical Stage**	Stage 4: n (%)	15 (18.75)	10 (23.81)		
	Stage 3: n (%)	25 (31.25)	16 (38.09)		
	Stage 2: n (%)	18 (22.50)	7 (16.67)		
	Stage 1: n (%)	22 (27.50)	9 (21.43)		

**Grade (Differentiation)**	Well: n (%)	46 (57.50)	25 (59.52)		
	Mod: n (%)	20 (25.00)	7 (16.67)		
	Poor: n (%)	14 (17.50)	10 (23.81)		

* The M stage data is not available.

### Immunohistochemistry analysis

Tissue samples were dehydrated in an ethanol series, cleared in xylene, and embedded in paraffin. Five-micrometer sections were prepared and mounted on poly-L-lysine-coated slides. Representative sections were stained with H&E and histologically evaluated by a pathologist. Immunohistochemical analysis was done using a commercially available kit (Invitrogen, Carlsbad, CA). Sections were incubated at 60°C for 30 min and deparaffinized in xylene. Endogenous peroxidase activity was quenched by incubation in a 9:1 methanol/30% hydrogen peroxide solution for 10 min at room temperature. Sections were rehydrated in PBS (pH 7.4) for 10 min at room temperature. Sections were then blocked with 10% normal serum for 10 min at room temperature followed by incubation with anti-Hsp27 and anti-Ki67 antibodies (ABCam) at a dilution of 1:200 for 16 h at room temperature. After washing thrice in PBS, the sections were incubated with secondary antibody conjugated to biotin for 10 min at room temperature. After additional washing in PBS, the sections were incubated with streptavidin-conjugated horseradish peroxidase enzyme for 10 min at room temperature. Following final washes in PBS, antigen-antibody complexes were detected by incubation with a hydrogen peroxide substrate solution containing aminoethylcarbazole chromogen reagent. Slides were rinsed in distilled water, coverslipped using aqueous mounting medium, and allowed to dry at room temperature. The relative intensities of the completed immunohistochemical reactions were evaluated using light microscopy by 3 independent trained observers who were unaware of the clinical data. All areas of tumor cells within each section were analyzed. All tumor cells in ten random high power fields were counted. A scale of 0 to 3 was used to score relative intensity, with 0 corresponding to no detectable immunoreactivity and 1, 2, and 3 equivalent to low, moderate, and high staining, respectively. As for Ki67, nuclear staining was evaluated and the Ki67 index was calculated as the percentage of positive stained nuclei of all the tumor cells in ten randomly selected high power fields.

### Statistical analysis

Spearman Correlation Coefficient was used to assess correlations among the gene expression and clinical and histopathological parameters. One-way ANOVA was used to assess the association of gene expression (e.g., Hsp27 and Ki67) with histopathological parameters (e.g., grading). Kaplan-Meier plots were constructed to present the survival outcomes of high Hsp27 cases (moderate to high staining) and low Hsp27 cases (no or low staining). Cox regression was used for both univariate and multivariate analysis. For multivariate analysis, age, gender, pathological T-stage (pT), pathological N-stage (pN), and clinical stages, grade, Ki67 index, and Hsp27 expression were considered as co-variates. For all statistical analyses, p < 0.05 was considered statistically significant.

## Results

**Figure 1:**
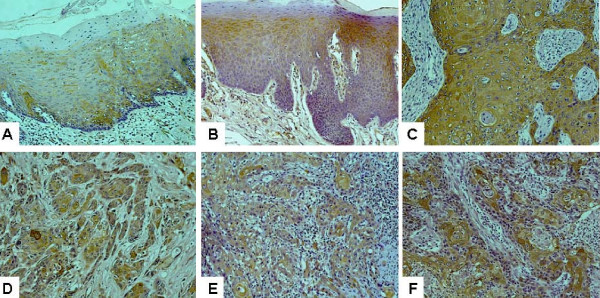
**Immunohistochemistry analyses of Hsp27 expression on normal tongue, dysplastic lesions, primary oral tongue squamous cell carcinoma (OTSCC) and lymph node metastasis tissue samples**. Immunohistochemistry analyses for Hsp27 were performed as described in Material and Methods on **A**: normal tongue mucosa (n = 15), **B**: dysplastic lesions (n = 31), **C**: well differentiated primary OTSCC (n = 46), **D**: moderately differentiated primary OTSCC (n = 20), **E**: poorly differentiated primary OTSCC (n = 14), and **F**: lymph node metastasis (n = 32). Representative Images (×200) were shown.

As illustrated in Figure 1, predominantly cytoplasmic staining for Hsp27 was observed in suprabasal keratinocytes in normal mucosa (Figure 1A). In general, the basal keratinocytes were not immunolabelled by anti-Hsp27 antibody. Among 15 normal mucosa that we examined, 4 cases (26.67%) showed no Hsp27 staining, 8 cases (53.33%) showed low staining, 2 cases (13.33%) showed moderate staining, and 1 case (6.67%) showed high staining. In dysplastic lesions (Figure 1B), the Hsp27 staining was observed in both suprabasal and basal keratinocytes. Of the 31 dysplastic lesions, 1 case (3.23%) showed no Hsp27 staining, 5 cases (16.13%) showed low staining, 11 cases (35.48%) showed moderate staining, and 14 cases (45.16%) showed high staining. In SCC (Figure 1C, D, E), diffuse Hsp27 staining was observed, where the high Hsp27 expression appears to be associated with differentiated areas. Of the 80 cases of primary OTSCC cases, 1 case (1.25%) showed no Hsp27 staining, 20 cases (25%) showed low staining, 42 cases (52.5%) showed moderate staining, and 17 cases (21.25%) showed high staining. For the 32 cases of lymph node metastatic disease (Figure 1F), low staining was observed in 8 cases (25%), moderate staining was observed in 12 cases (37.5%), and high staining was observed in 12 cases (37.5%). Thus, the dysregulation of Hsp27 consists of both the changes in cell population expressing this gene and the alterations in expression level. This change in Hsp27 expression pattern is consistent with our previous observations [22].

Correlations were tested among gene expression (e.g., Hsp27 and Ki67), clinical and Hsp27 expression (Table 2). As expected, strong correlations were observed among pT, pN, and Clinical stage. While correlation between Ki67 index and grading was observed (p < 0.05), a significant inverse correlation was observed between Hsp27 expression and grading (p < 0.01). Interestingly, correlation between Hsp27 expression and age was also observed (p < 0.05).

**Table 2:** Correlations among clinical and histopathological features of primary OTSCC*

	Age	Gender	pT stage	pN stage	C stage	Grade	Ki67	Hsp27
**Age**		0.01	-0.04	-0.13	-0.06	-0.10	0.18	0.26 **
**Gender**			0.12	0.04	0.12	0.00	0.07	-0.03
**pT stage**				0.44 ***	0.81 ***	-0.14	-0.14	0.00
**pN stage**					0.78 ***	0.06	-0.03	0.16
**C stage**						-0.01	-0.07	0.06
**Grade**							0.23 **	-0.29 ***
**Ki67**								0.04
**Hsp27**								

*Spearman Correlation Coefficients were presented. pT: pathological T-stage; pN: pathological N-stage.

** p < 0.05

*** p < 0.01

As illustrated in Figure 1C, D, and 1E, the higher expression of Hsp27 was observed in well and moderately differentiated OTSCC, and lower Hsp27 expression was observed in poorly differentiated cases. Statistical analysis revealed that the Hsp27 expression level in primary OTSCC is associated with differentiation (p = 0.0259) (Table 3). A marginal association was also observed between Ki67 index and differentiation (p = 0.0613).

**Table 3:** Association of Hsp27 expression and Ki67 index with differentiation*

		Hsp27 (IHC)		Ki67 index	
		
	n	Average	Variance	Average	Variance
**Well**	46	1.90	0.45	0.225	0.019
**Moderately**	20	1.74	0.47	0.302	0.025
**Poorly**	14	1.34	0.39	0.316	0.040
			(p = 0.025872)		(p = 0.061275)

*One-way ANOVA was used to assess the association of Hsp27 and Ki67 with grading.

**Figure 2:**
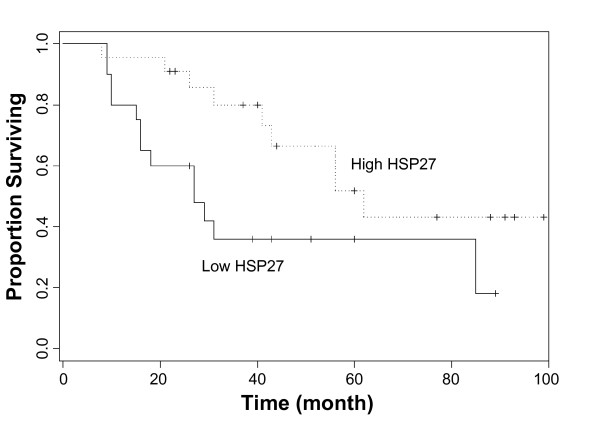
**The effects of Hsp27 expression on prognosis**. Kaplan-Meier plots of overall survival in patient groups defined by Hsp27 immunohistochemistry (High Hsp27: moderate to high staining, Low Hsp27: no detectable or low staining). The difference in survival rates is statistically significant (p < 0.05).

Among 80 cases of OTSCC that we examined, follow-up results were available on 42 cases (52.5%). Median duration of follow-up was 31 months (range 8–99). Among these 42 cases, 12 cases (28.57%) showed low Hsp27 staining, 18 cases (42.86%) showed moderate Hsp27 staining, and 12 cases (28.57%) showed high Hsp27 staining. As illustrated in Figure 2, a striking difference in median survival time was observed between high (immunohistochemical score of 2 or 3) Hsp27 expression group (over 60 months) and low (immunohistochemical score of 0 or 1) Hsp27 expression groups (less than 30 months). Both univariate and multivariate analysis demonstrated the positive effect of high Hsp27 expression for prognosis (Table 4). Based on univariate analysis, the effects of pT and clinical stages on prognosis were also observed.

**Table 4:** The effects of clinical and pathohistological parameters on prognosis*

	Univariate Analysis		Multivariate Analysis	
	
	HR (95% CI)	p value	HR (95% CI)	p value
**Age**	0.991 (0.958, 1.025)	0.5941		
**Gender**	1.991 (0.849, 4.670)	0.1135		
**pT**	**1.622 (1.032, 2.551)**	**0.0362**	2.565 (0.924, 7.121)	0.0706
**pN**	1.514 (0.893, 2.564)	0.1233		
**C Stage**	**1.599 (1.028, 2.485)**	**0.0371**	0.679 (0.179, 2.568)	0.5680
**Grade**	1.501 (0.889, 2.534)	0.1291		
**Ki67**	0.547 (0.034, 8.761)	0.6698		
**Hsp27**	**0.375 (0.190, 0.741)**	**0.0048**	**0.234 (0.091, 0.599)**	**0.0025**

* Analysis was done with Cox proportional hazard regression. HR: hazard ratio; 95% CI: 95% confidence interval.

## Discussion and conclusion

Heat shock proteins (HSPs) have been implicated in multiple aspects of tumorigenesis [29]. Hsp27 belongs to a sub-family of small heat shock proteins that are characterized by low molecular masses (12–43 kDa). High levels of Hsp27 expression have been observed in many types of cancers, and tumorigenic potential of Hsp27 expression has been observed in experimental models [30,31]. Its antiapoptotic effect has been well established [32]. The dysregulation of Hsp27 has also been suggested to contribute to invasion and metastasis [33]. More importantly, Hsp27 expression is associated with resistance to cytostatic anticancer drugs (e.g., cisplatin, vincristine and colchicine) and radiation therapy [34-37]. It is striking that Hsp27 is associated with poor prognosis in several tumor types (gastric [12,13], liver [14] and prostate carcinoma [15]), but good prognosis in others (endometrial adenocarcinomas [16,17] and oesophageal cancer [18,19]). These apparent differences in the prognosis of Hsp27 may be partly due to the difference in biological natures of these cancer types, as well as the different treatment options (some of them may be influenced by Hsp27) for these cancers. Collectively, these facts signify the profound roles of Hsp27 in many aspects of tumor progression and response to therapy.

Currently, the prognostic significance of Hsp27 for OSCC is not clear. While correlation of Hsp27 expression with survival rate was observed in some studies [24,25,28], inverse correlation with survival was implicated in a different study [27], and no prognostic significance was observed by other studies [23,26]. These inconsistencies may in part be due to the heterogeneity in oncogenic pathways. Distinct oncogenic pathways may give rise to a common malignant phenotype (i.e. there may be several different "ways" to get oral cancers). If there are multiple distinct etiologic possibilities (e.g., tobacco smoking, alcohol abuse, HPV infection), and not all are necessary to produce the malignant phenotype, then some of the oncogenic pathways may remain "normal" despite the fact that cancer develops. This may be especially true for OSCC, which arises in various sites (e.g., oral cavity and oropharynx). Tumors from these different sites have distinct clinical presentations and clinical outcomes, and are associated with different risk factors [1] and genetic characteristics [2]. This again complicates the evaluation of prognostic markers because no single etiologic event is consistently present in all cases.

In our present study, by focusing on just one anatomic site (oral tongue), we were able to partially minimize the genetic variation and microenvironment effects among intraoral sites. Our results clearly demonstrated that higher expression level of Hsp27 in primary OTSCC is directly correlated with overall survival rate. Our results also confirmed that the dysregulation of Hsp27 expression is a progressive event that accompanies the OTSCC progression. While the Hsp27 expression is restricted to differentiated suprabasal keratinocytes in normal mucosa, the Hsp27 is expressed in both suprabasal and basal keratinocytes of dysplastic lesions, and then exhibits a diffuse expression pattern in primary OTSCC and metastatic disease. In addition, the reduced Hsp27 expression in primary tumor tissue is associated with poor differentiation. Interestingly, correlation between Hsp27 expression and age was also observed (p < 0.05). A similar observation regarding age and Hsp27 expression in OSCC was also reported in a previous study [24]. Currently, the clinical relevance of this observation is not clear.

It should be noticed that the biological activities of Hsp27 are regulated by phosphorylation of its serines by mitogen-activated protein kinases associated protein kinases (MAPKAPKs) which are themselves activated by phosphorylation by MAP p38 protein kinase [38,39]. In our present study, we do not know the phosphorylation status of the Hsp27. Additional studies may be warranted to further evaluate the prognostic value of Hsp27 which incorporates additional stratification of the phosphorylation status.

In summary, we demonstrated that the dysregulation of Hsp27 expression is a frequent event during the progression of OTSCC. The expression level of Hsp27 is associated with differentiation grade, and reduced expression of Hsp27 is correlated with poor overall survival. Our data suggested that Hsp27 expression may provide prognostic value for patients with OTSCC.

## Competing interests

The authors declare that they have no competing interests.

## Authors' contributions

AW, HY, and XZ conceived the idea for the project and drafted the manuscript. AW, XL, SS, HY and TP performed the laboratory analyses. FS and XZ conducted statistical analyses. AW, XL and DC provided discussions on clinical relevance. AW, XL, DC and XZ drafted the manuscript. All authors read and approved the final manuscript.

## Pre-publication history

The pre-publication history for this paper can be accessed here:

http://www.biomedcentral.com/1471-2407/9/167/prepub
